# Suboptimal Tongue Pressure Is Associated with Risk of Malnutrition in Community-Dwelling Older Individuals

**DOI:** 10.3390/nu13061821

**Published:** 2021-05-27

**Authors:** Ke-Vin Chang, Wei-Ting Wu, Lan-Rong Chen, Hsin-I Wang, Tyng-Guey Wang, Der-Sheng Han

**Affiliations:** 1 Department of Physical Medicine and Rehabilitation, National Taiwan University Hospital, Bei-Hu Branch, Taipei 108, Taiwan; kvchang011@gmail.com (K.-V.C.); wwtaustin@yahoo.com.tw (W.-T.W.); lchen@livemail.tw (L.-R.C.); cindy8912@gmail.com.tw (H.-I.W.); 2 Department of Physical Medicine and Rehabilitation, National Taiwan University College of Medicine, Taipei 100, Taiwan; tgw@ntu.edu.tw; 3 Center for Regional Anesthesia and Pain Medicine, Wang-Fang Hospital, Taipei Medical University, Taipei 117, Taiwan; 4 Health Science and Wellness Center, National Taiwan University, Taipei 106, Taiwan

**Keywords:** malnutrition, dysphagia, sarcopenia, oral frailty, tongue pressure

## Abstract

The tongue plays an important role in swallowing, and its dysfunction theoretically leads to inadequate oral intake and subsequent malnutrition. This study aimed to explore how different levels of tongue pressure are related to malnutrition among community-dwelling older individuals. The target population was community-dwelling adults aged ≥ 65 years. Tongue pressure was measured using the Iowa Oral Performance Instrument, whereas the mini nutrition assessment (MNA) test was administered to determine the nutritional status. A full MNA score of less than 24 points was defined as risk of malnutrition. Multivariate logistic regression analyses were conducted to calculate the odds ratio (OR) of risk of malnutrition among different quartiles of tongue pressure. Among the 362 participants, 26 (7.1%) were classified as having risk of malnutrition. Body weight, body mass index, handgrip strength, skeletal muscle mass index, sum MNA score, and serum levels of albumin were lower in the malnutrition risk groups than in the normal nutrition status group. A positive correlation was identified between tongue pressure and the MNA score (*r* = 0.143, *p* < 0.01). Treating the subgroup of the highest quartile of tongue pressure as the reference, the crude odds ratio (OR) of having risk of malnutrition was 5.37 (95% CI, 1.14–25.28) in the subgroup at the third quartile, 3.10 (95% CI, 0.60–15.84) in the subgroup at the second quartile, and 3.95 (95% CI, 0.81–19.15) in the subgroup at the lowest quartile. After adjustment for age and sex, the subgroup in the third quartile still presented with a significantly higher risk (OR, 4.85; 95% CI, 1.02–22.99) of risk of malnutrition. Compared with the subgroup at the highest quartile of tongue pressure, the crude OR for all the subgroups in the lower three quartiles was 4.17 (95% CI, 0.96–18.04), showing borderline significance (*p* = 0.05). In conclusion, we found hints for an association between decreased tongue pressure and an increased risk of malnutrition in community-dwelling older individuals. Older people with suboptimal tongue pressure should undergo a thorough assessment of their nutritional status and swallowing function for the early identification of subclinical malnutrition and dysphagia.

## 1. Introduction

The impact of aging on oral health has recently been recognized [1]. Older adults have a higher risk than younger people to suffer from missing teeth, dental caries, periodontitis, and intra-oral malignancy [1]. The decrease in skeletal muscle mass and function, including those for mastication and swallowing, has emerged as a medical issue in the geriatric population, leading to a rising number of comorbidities and impaired quality of life [2]. The tongue, including its intrinsic and extrinsic muscles, predominantly encompasses type II muscle fibers [3] and can be affected by malnourishment and various neurological disorders, such as strokes [4] and parkinsonism [5]. The age-related decline in tongue pressure has been uncovered recently, and a cross-sectional study demonstrated a negative correlation between tongue thickness and age [6]. The tongue plays an unneglectable role in swallowing, especially during the oral preparatory phase, and its dysfunction theoretically leads to an inadequate oral intake and subsequent malnutrition.

Oral frailty, defined as a decline in oral health related to changes in swallowing due to aging, has been found to be associated with malnutrition. In 2020, Iwasaki et al. enrolled 1454 Japanese community-dwelling elders to assess their oral function and nutritional status [7]. The study revealed that participants with oral frailty had an increased risk of severe malnutrition, as determined by the short form of the mini nutrition assessment (MNA) questionnaire. The cause of oral frailty is multifaceted, encompassing tooth loss, dental caries, periodontal disease, oral motor skill impairment, decreased masticatory performance, and weakened swallowing muscles [1]. In terms of the functional evaluation of the swallowing muscles, the widely used approach is to measure tongue pressure [8]. An antecedent study demonstrated acceptable reliability of tongue pressure measurement using the Iowa Oral Performance Instrument, especially when the examiners were familiar with the measuring protocol [9]. Considering tongue pressure as a reliable assessment of oral function, it would be of clinical importance to determine the association between tongue pressure and malnutrition.

Older people are vulnerable to malnutrition, especially those with chronic diseases or those living in long-term care facilities. The prevalence of malnutrition in community-dwelling residents has been reported to be 3.1%, and that in geriatric hospitalized patients can be as high as 22% [10]. Elders are likely to develop dental problems, making them to refrain from harder foods (such as unprocessed meat, fruits, and vegetables), which are abundant in proteins, fibers, vitamins, and minerals [11]. Furthermore, the aging population prefers soft foods that contain higher levels of sugar and fat and lower levels of micronutrients, eliciting an increase in oxidative stress and chronic inflammation [11]. Consequently, malnutrition has been shown to be associated with several adverse health outcomes, including longer hospital admission, more post-surgical complications, and higher in-hospital mortality [12]. Recently, the strength of tongue muscles has been considered an important indicator of oral health [13], which plays a crucial role in nutrition intake. Until now, there has been a lack of large-scale studies investigating the association between tongue pressure and nutritional status in the aging group. This study aimed to explore how different levels of tongue pressure are related to malnutrition among community-dwelling elders.

## 2. Materials and Methods

### 2.1. Participants

The target population of the present study was community-dwelling older adults aged ≥ 65 years who underwent a yearly health checkup in a community hospital in Taipei, Taiwan. The hospital is located in Wan-Hwa district of Taipei city, where the average age of the residents is older than that in other districts. The percentage of retired old people is also higher in Wan-Hwa district. The main patient source of the hospital is the residents living in the nearby communities. Participants older than 65 years are entitled to receive free annual check-up, including history taking, physical examinations, urine analysis, and routine serum biochemistry. In this study, the exclusion criteria were: (1) the inability to walk independently without using devices; (2) cognitive impairment; (3) failure to understand verbal instructions and respond to the questionnaire; (4) undergoing treatments for active oncological problems; and (5) having a poorly managed chronic disease (such as hyperosmolar hyperglycemic non-ketoacidosis and unstable angina). In the present study, the AD-8 (dementia screening interview) was used to identify participants with potential cognitive impairment [14]. A score equal to or larger than 2 was deemed abnormal. The Institutional Review Board of the National Taiwan University Hospital approved this study (IRB No. 201812037RIND). Written informed consent was obtained from all the participants before enrollment. The study period was from March 2019 to September 2019 and 366 participants were screened with 4 people excluded from the final enrollment mainly due to inability to walk independently without using devices.

### 2.2. Biochemical Measurements

Venous blood samples were collected from the participants after overnight fasting for at least 8 h. The values of albumin were measured using an auto-analyzer (Hitachi 7250 Special, Hitachi, Tokyo, Japan) [15].

### 2.3. Measurement of Body Composition

An InBody 270 multi-frequency body composition analyzer (Biospace, Seoul, Korea) was employed to measure the body composition using a tetra-polar 8-point tactile electrode system [16]. During the examination, the participant stood upright on the foot electrodes of the main operation unit without footwear and gripped the two hand electrode holders firmly with both hands. Prior to the test, the participants were asked to use alcohol swabs to clean both the palms and soles. Due to the fact that bioimpedance analysis is vulnerable to the influence of the hydration status [17], participants were required not to consume alcohol or perform intense exercise for 24 h before the measurement.

### 2.4. Assessment of Physical Performance

When measuring grip strength, the participants were seated with their elbow in 90 degrees of flexion. They were required to squeeze the isometric dynamometer (Baseline^®^ hydraulic hand dynamometer, Fabrication Enterprises Inc., Irvington, NY, USA) with maximal efforts three times by using their dominant hands. The unit of grip strength measurement was kg. An interval of at least 1 min between each trial was required to prevent the tested hands from fatigue. The maximum value among the three attempts was used for analysis [18]. 

Regarding the measurement of gait speed, four markers were attached to a straight line on the ground at the starting points, 1, 6, and 7 m. The gait speed (m/s) was calculated using the following formula: 5/time in seconds from the 1-m to 6-m points [18]. Participants were informed to ambulate at a comfortable speed. 

### 2.5. Definition of Sarcopenia

Sarcopenia was diagnosed by fulfilling the criteria of low muscle mass and function using the consensus of the European Working Group on Sarcopenia in Older People (EWGSOP1) [19]. Low muscle function was defined as grip strength of less than 30 kg in men and 20 kg in women. Low muscle mass indicated a skeletal muscle mass index less than 7.40 kg/m^2^ for men and 5.14 kg/m^2^ for women. In the present study, we did not use EWGSOP2 [20] for concern of comparability with other diagnostic algorithm for sarcopenia [21] and consistency with our serial studies [22,23,24]. 

### 2.6. Tongue Pressure

Maximal tongue pressure was measured using the Iowa Oral Performance Instrument^®^ (IOPI, Northwest Co., LLC, Carnation, WA, USA) [25]. The IOPI contains an air-filled bulb, a silicon tube, and a processing instrument to display the contact pressure values. The air-filled bulb was placed between the tongue and the anterior portion of the hard palate by the same examiner. The silicon tube was positioned along the midline of the upper lip and secured by approximating the upper and lower incisors. The examinees were informed not to bite the tube forcefully and were asked to compress the bulb with maximal effort three times. The minimal interval between each compression attempt was 1 min. The maximum value among the three trials was used for the analysis. The measurement was performed by the same speech therapist (H.-I.W.) and the unit of tongue pressure measurement was kPa.

### 2.7. Evaluation of Malnutrition

The MNA test was administered to identify participants with malnutrition or at risk of malnutrition [26]. The dimensions assessed in the MNA test encompassed anthropometric measurements (body mass index, weight loss, and the circumferences of the calves and mid-arms), assessment of oral intake (amount of protein, fruit, vegetable, fluid and overall food consumption, number of daily full meals, mode of feeding, and medication ingestion), self-perception of nutritional and health status, and psychomotor well-being (recent presence of psychological stress or acute illness, neuropsychological problems, independent living, and existence of pressure ulcer). The maximum sum score of the full MNA test was 30 points. A score of 24 points or more was considered adequate nutrition status, a score between 17 and 23.5 points was considered at risk of malnutrition, and a score of less than 17 points was considered malnourished. In the present study, we defined an MNA score of less than 24 points as risk of malnutrition.

### 2.8. Evaluation of Swallowing Function

Swallowing function was evaluated using Eating Assessment Tool (EAT)-10, a self-administered, symptom-specific outcome instrument for screening dysphagia. The EAT-10 encompasses 10 queries rated on a 5-point Likert scale from no problem (0 point) to severe problem (5 points), with a sum score ranging from 0 to 40. A sum score ≥ 3 was considered a risk factor for dysphagia [27].

### 2.9. Statistical Analysis

The participants were divided into quartiles based on their tongue-pressure values. Continuous variables are shown as the mean ± standard deviation, and categorical variables are presented as number (percentage). The Shapiro-Wilk test was used to determine whether the data were normally distributed. Continuous variables were compared using the Student’s *t*-test or Mann-Whitney U test (for non-normally distributed data). Categorical variables were compared using the chi-square test or Fisher’s exact test (in case of sparse data). Multivariate logistic regression analyses were conducted to calculate the odds ratio (OR) and 95% confidence interval (CI) of risk of malnutrition (the sum score of the MNA test ≤ 23.5) among different quartiles of tongue pressure following adjustment for age, sex, skeletal muscle mass index, grip strength, and gait speed. The ORs of the lower three quartiles of tongue pressure versus those in the highest quartile were also analyzed by using the multivariate logistic regression. Pearson’s correlation coefficient (r) was used to measure the correlation between tongue pressure and skeletal muscle mass index, grip strength, and gait speed. Statistical analyses were performed using SPSS statistical software (V.17, SPSS, Chicago, IL, USA). Statistical significance was set at *p* < 0.05.

## 3. Result

### 3.1. Participant Characteristics

Overall, 213 women (58.84%) and 149 men (41.16%) were included in the present study, with an average age of 71.75 ± 5.29 years (95% CI, 71.20–72.30). The basic characteristics of the participants are shown in Table 1, including 26 older individuals with an MNA score ≤ 23.5 points (risk of malnutrition) and 336 persons with an MNA score ≥ 24 points (adequate nutrition). Among the former group, only one participant had an MNA score of <17 points. Body weight, body mass index, handgrip strength, skeletal muscle mass index, sum MNA score, and serum levels of albumin were lower in the malnutrition risk groups than in the normal nutrition status group. The sum EAT-10 score and prevalence of risk for dysphagia (EAT-10 score ≥ 3) and sarcopenia were also higher in the former group than in the latter group.

### 3.2. Association of Risk of Malnutrition with Tongue Pressure

**Figure 1:**
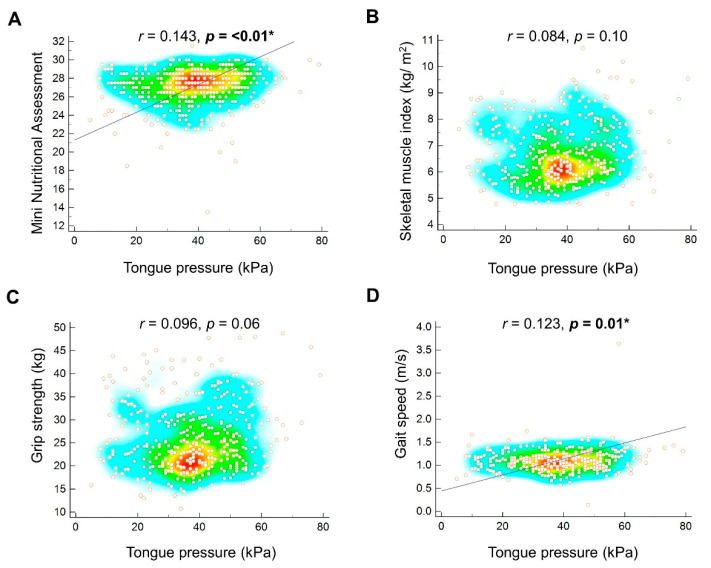
Correlation of tongue pressure with the mini nutritional assessment (**A**), skeletal muscle mass index (**B**), grip strength (**C**), and gait speed (**D**). The regression line is plotted and * is marked when *p* value is less than 0.05. The heat map with the background color coding suggests clusters of observations.

A positive correlation was identified between tongue pressure and the MNA score (*r* = 0.143, *p* < 0.01) (Figure 1A). We further divided our study participants into four subgroups based on the quartiles of tongue pressure. Compared with the subgroup with the highest quartile of tongue pressure, the other three subgroups were likely to have a higher proportion of participants with malnutrition and risk of malnutrition. Treating the subgroup of the highest quartile of tongue pressure as the reference, the crude OR of having risk of malnutrition was 5.37 (95% CI, 1.14–25.28) in the subgroup at the third quartile, 3.10 (95% CI, 0.60–15.84) in the subgroup at the second quartile, and 3.95 (95% CI, 0.81–19.15) in the subgroup at the lowest quartile. After adjustment for age and sex, the subgroup in the third quartile still presented with a significantly higher risk (OR, 4.85; 95% CI, 1.02–22.99) of risk of malnutrition. However, the statistical significance for the subgroup in the third quartile diminished after the addition of skeletal muscle mass index, grip strength, and gait speed in the adjustment (OR, 3.82; 95% CI, 0.77–18.76) (Table 2). The OR of risk of malnutrition did not show a gradual increase as the tongue pressure decreased (*p* for trend > 0.05, in all three regression models). 

Compared with the subgroup at the highest quartile of tongue pressure, the crude OR for the subgroups in the lower three quartiles was 4.17 (95% CI, 0.96–18.04), showing borderline significance (*p* = 0.05). The statistical significance diminished after adjustment for age and sex (OR, 3.83; 95% CI, 0.88—16.65; *p* = 0.07) and both above-mentioned factors plus skeletal muscle mass index, grip strength, and gait speed (OR, 2.64; 95% CI, 0.58–11.82; *p* = 0.20) (Supplemental Table S1).

### 3.3. Correlation between Tongue Pressure and Muscle Mass and Function

The correlation analysis revealed insignificant correlations between tongue pressure and skeletal muscle mass index (*r* = 0.084, *p* = 0.10) (Figure 1B) and between tongue pressure and grip strength (*r* = 0.096, *p* = 0.06) (Figure 1C). Tongue pressure was positively correlated with gait speed (*r* = 0.123, *p* = 0.01) (Figure 1D).

### 3.4. Subgroup Analysis According to Sex

A subgroup analysis was performed in accordance with different sex groups. Treating the subgroup of the highest quartile of tongue pressure as the reference, the associations between tongue pressure lower than the upper quartile and the risk of malnutrition were presented in Table 3 and Table 4. The direction of the point estimates of OR in each subgroup was consistent in the female (Table 3) and male participants (Table 4).

## 4. Discussion

The association between tongue pressure and malnutrition in community-dwelling older individuals was investigated in this study, contributing to several important findings. First, the risk of malnutrition was increased in the groups with lower quartiles of tongue pressure compared with those in the highest quartile. Second, tongue pressure was positively correlated with gait speed. Third, the direction of the association between tongue pressure and risk of malnutrition did not differ between the female and male subgroups.

In the present study, the risk of malnutrition was higher in the groups with lower quartiles of tongue pressure compared with those in the highest quartile. The association between decreased tongue pressure and malnutrition may be bidirectional. Malnutrition is a widely known risk factor for sarcopenia [28] and is characterized by loss of muscle mass and function. Tongue pressure, as a surrogate indicator of tongue strength, may be reduced in accordance with the change of grip strength in the sarcopenic population [29]. In contrast, the motor performance of the tongue is closely related to the swallowing function, especially during the oral preparatory phase [30]. A decline in tongue pressure would significantly hinder food bolus formation and propagation, thus leading to malnourishment following decreased oral intake. However, as the present study utilized a cross-sectional design, it remains challenging to determine the causal relationship between malnutrition and low tongue pressure.

In our data, the upper quartile of tongue pressure was 48 kPa, which was higher than the threshold (21.6 kPa) of low tongue pressure proposed by Nakamori et al. [31]. The participants enrolled in the aforementioned study were 220 acute stroke patients, whose basic characteristics were likely to be different from community-dwelling older people. Therefore, our analysis implied that a suboptimal level of tongue pressure was independently associated with risk of malnutrition. In addition, we did not identify a trend of a gradual increase in the risk of malnutrition from the participants in the 3rd quartile to those in the 1st quartile. This finding might be attributed to the multiple causes of malnutrition (e.g., impaired swallowing, poor oral intake, unbalanced diet, and chronic disease), and decreased tongue pressure is merely a risk factor [12]. In this regard, a linear relationship between the risk of malnutrition and decreased tongue pressure was not observed in our analysis, shown on the hypothesis testing for trend (dose-response effect in association). All the *p* values for trend were larger than 0.05 (Table 2).

Our data revealed that tongue pressure was correlated with an increase in gait speed. Our findings were consistent with those of a previous study including 54 elderly participants living in a nursing home that demonstrated a positive relationship between tongue pressure and grip strength (*r* = 0.291) and gait speed (*r* = 0.408) [32]. The magnitude of association between tongue pressure and physical performance in the aforementioned study seemed to be larger than ours. We speculated that the finding might be attributed to worse swallowing function in their participants, with an average of 2.37 points on EAT-10. However, our study showed no significant association between tongue pressure and skeletal muscle mass. This observation implies that a decrease in extremity muscle strength and function might result from an impact on the skeletal muscles of the whole body, and that the tongue muscles might be affected in the systemic process. Therefore, if a participant is diagnosed with sarcopenia, tongue pressure, and nutritional status should be examined for early recognition of subclinical dysphagia and malnutrition.

A subgroup analysis was performed to determine the influence of sex on the association between tongue pressure and malnutrition, which revealed consistency in the direction of association in female and male participants. In our study, we used sex-specific analyses, with separate quartile values applied on the female and male subgroups (Table 3 and Table 4). A previous study defined the threshold of low tongue pressure according to sex (men < 27.4 kPa and women < 26.5 kPa) [13], using a scenario similar to that for determining sarcopenia. Nevertheless, Nicosia et al. reported a lack of sex differences in isometric and peak swallowing pressure estimated by intraoral pressure sensors in a population without swallowing dysfunction [33]. Similar to the study done by Nicosia et al. [33], the sex difference in tongue pressure would be theoretically minimal in our study, as we investigated relatively healthy community-dwelling elders. Even though an undetected sex difference existed, its impact was adjusted in our regression analysis, which further validated the actual association between tongue pressure and malnutrition.

Some limitations of this study need to be acknowledged. First, this was a cross-sectional study. The causal relationship between tongue pressure and malnutrition should be explored using a prospective cohort study. Second, only one case had an MNA score of less than 17 points, indicating that the prevalence of definite malnutrition was extremely low in our study participants. Therefore, if we intend to explore the association between tongue pressure and definite malnutrition, the target population might be focused on long-term care facility residents. Third, the swallowing function could not be totally represented by tongue pressure, as the main role of the tongue is in the oral preparatory phase. In the present study, the EAT-10 was used as an additional assessment for swallowing performance, which revealed a higher prevalence of swallowing dysfunction in the group with risk of malnutrition. Fourth, we did not record whether the participants had depressive symptoms or the medication they took, which might confound the outcome and are suggested to be included in the subsequent study. Fifth, the number of cases with risk of malnutrition in each subgroup was relatively small. This would influence the precision of effect size measurement, causing an increase in uncertainty and enlargement of the 95% confidence interval. Therefore, more precaution is needed for interpreting the result from logistic regression analysis. Sixth, the healthy worker effect might be the reason why only a few participants (*n* = 26) were classified as risk of malnutrition. The old participants who care more about their health were more likely to attend the annual health check-up. Therefore, the actual prevalence of malnutrition in the community-dwelling elders might be underestimated in our study. Seventh, we did not document the number of teeth, dental status and person’s ability to chew, swallow, and eat. The above mentioned information pertinent to oral health should be detailed in future research.

## 5. Conclusions

We found hints for an association between decreased tongue pressure and an increased risk of malnutrition in community-dwelling older individuals. Older people with suboptimal tongue pressure should undergo a thorough assessment of the nutritional status and swallowing function for the early identification of subclinical malnutrition and dysphagia. A prospective cohort study and a randomized controlled trial are warranted to investigate the causal relationship between tongue pressure and malnutrition and how these conditions interact in long-term care facility residents.

## Figures and Tables

**Table 1:** Characteristics of the study participants.

	Risk of Malnutrition (*n* = 26)	Normal Nutritional Status (*n* = 336)	*p* Value
Age (years)	73.53 ± 6.33	71.61 ± 5.18	0.12
	(70.98 to 76.09)	(71.06 to 72.17)	
Female (%)	17 (65.38%)	196 (58.33%)	0.48
	(45.78% to 84.98%)	(53.03% to 63.63%)	
Height (cm)	158.35 ± 10.89	158.89 ± 8.01	0.45
	(153.95 to 162.75)	(158.03 to 159.75)	
Weight (kg)	50.13 ± 10.39	59.03 ± 9.40	<0.01 *
	(45.93 to 54.32)	(58.02 to 60.04)	
Body mass index (kg/m^2^)	19.85 ± 2.70	23.33 ± 2.90	<0.01 *
	(18.76 to 20.95)	(23.02 to 23.64)	
Skeletal muscle index (kg/m^2^)	6.13 ± 1.20	6.80 ± 1.11	<0.01 *
	(5.64 to 6.62)	(6.68 to 6.92)	
Grip strength (kg)	22.88 ± 6.80	26.52 ± 7.33	<0.01 *
	(20.13 to 25.63)	(25.73 to 27.31)	
Sarcopenia (%)	10 (38.46%)	23 (6.84%)	<0.01 *
	(18.42% to 58.50%)	(4.13% to 9.55%)	
Gait speed (m/s)	1.05 ± 0.18	1.10 ± 0.24	0.29
	(0.98 to 1.13)	(1.07 to 1.13)	
Mini Nutritional Assessment	22.01 ± 2.24	27.40 ± 1.49	<0.01 *
	(21.11 to 22.92)	(27.24 to 27.56)	
EAT-10	1.92 ± 3.01	0.72 ± 1.47	0.01 *
	(0.70 to 3.14)	(0.56 to 0.88)	
Risk for dysphagia (%)	8 (30.76%)	23 (6.84%)	<0.01 *
	(11.75% to 49.78%)	(4.13% to 9.55%)	
Tongue pressure (kPa)	34.84 ± 11.57	38.20 ± 14.01	0.27
	(30.17 to 39.52)	(36.70 to 39.71)	
Albumin (g/dL)	4.11 ± 0.27	4.26 ± 0.22	<0.01 *
	(4.00 to 4.22)	(4.24 to 4.29)	
Cardiovascular disease (%)	1 (3.84%)	38 (11.30%)	0.23
	(−4.07% to 11.77%)	(7.90% to 14.71%)	
Diabetes mellitus (%)	2 (7.69%)	32 (9.52%)	0.75
	(−3.28% to 18.67%)	(6.36% to 12.68%)	
Hyperlipidemia (%)	4 (15.38%)	76 (22.61%)	0.39
	(0.52% to 30.25%)	(18.12% to 27.12%)	
Chronic kidney disease (%)	2 (7.69%)	7 (2.08%)	0.07
	(−3.28% to 18.67%)	(0.54% to 3.61%)	

The continuous data are shown as mean ± standard deviation (95% confidence interval) and compared by using the Student’s *t*-test or Mann-Whitney U test (for non-normally distributed data). The categorical variables are shown as number (percentage and 95% confidence interval) and compared using the chi-square test or Fisher’s exact test (in case of sparse data). EAT-10: Eating Assessment Tool-10. * *p* < 0.05.

**Table 2:** The association of risk of malnutrition with different quartiles of tongue pressure in the overall participants.

Risk of Malnutrition	Quartile of Maximum Tongue Pressure				*p* for Trend
	Q1 (*n* = 95)	Q2 (*n* = 89)	Q3 (*n* = 90)	Q4 (*n* = 88)	
	(≤29 kPa)	(30–38 kPa)	(39–48 kPa)	(>48 kPa)	
Number (percentage)	8	6	10	2	0.25
	(8.42%)	(6.74%)	(11.11%)	(2.27%)	
Model 1				1.00	0.18
OR	3.95	3.10	5.37 *		
(95% CI)	(0.81 to 19.15)	(0.60 to 15.84)	(1.14 to 25.28)		
Model 2				1.00	0.22
OR	3.82	2.72	4.85 *		
(95% CI)	(0.78 to 18.59)	(0.53 to 14.03)	(1.02 to 22.99)		
Model 3				1.00	0.26
OR	2.75	1.62	3.82		
(95% CI)	(0.54 to 13.88)	(0.30 to 8.78)	(0.77 to 18.76)		

OR, odds ratio. Model 1: no adjustment. Model 2: adjusted for age and sex. Model 3: adjusted for age, sex, skeletal muscle index, grip strength and gait speed. * *p* < 0.05.

**Table 3:** The association of risk of malnutrition with different quartiles of tongue pressure in the female participants.

Risk of Malnutrition	Quartile of Maximum Tongue Pressure				*p* for Trend
	Q1 (*n* = 54)	Q2 (*n* = 58)	Q3 (*n* = 53)	Q4 (*n* = 48)	
	(≤29 kPa)	(30–38 kPa)	(39–48 kPa)	(>48 kPa)	
Number (percentage)	6	4	6	1	0.20
	(11.11%)	(6.89%)	(11.32%)	(2.08%)	
Model 1				1.00	0.34
OR	5.87	3.48	6.13		
(95% CI)	(0.68 to 50.68)	(0.37 to 32.24)	(0.71 to 52.92)		
Model 2				1.00	0.34
OR	5.92	3.44	6.03		
(95% CI)	(0.68 to 51.52)	(0.36 to 32.19)	(0.69 to 52.49)		
Model 3				1.00	0.41
OR	2.95	1.83	4.67		
(95% CI)	(0.30 to 28.55)	(0.17 to 18.83)	(0.50 to 42.99)		

OR, odds ratio. Model 1: no adjustment. Model 2: adjusted for age. Model 3: adjusted for age, skeletal muscle index, grip strength and gait speed.

**Table 4:** The association of risk of malnutrition with different quartiles of tongue pressure in the male participants.

Risk of Malnutrition	Quartile of Maximum Tongue Pressure				*p* for Trend
	Q1 (*n* = 38)	Q2 (*n* = 37)	Q3 (*n* = 37)	Q4 (*n* = 37)	
	(≤28 kPa)	(29–39 kPa)	(40–49 kPa)	(>49 kPa)	
Number (percentage)	2	3	3	1	0.66
	(5.26%)	(8.10%)	(8.10%)	(2.70%)	
Model 1				1.00	0.72
OR	1.99	3.37	3.17		
(95% CI)	(0.17 to 23.04)	(0.33 to 34.09)	(0.31 to 32.03)		
Model 2				1.00	0.82
OR	1.95	2.88	2.81		
(95% CI)	(0.16 to 22.58)	(0.27 to 30.69)	(0.27 to 29.23)		
Model 3				1.00	0.94
OR	1.95	1.55	2.02		
(95% CI)	(0.16 to 23.54)	(0.13 to 18.02)	(0.18 to 22.64)		

OR, odds ratio. Model 1: no adjustment. Model 2: adjusted for age. Model 3: adjusted for age, skeletal muscle index, grip strength and gait speed.

## Data Availability

The data presented in this study are available on request from the corresponding author.

## Supplementary Materials

The following are available online at https://www.mdpi.com/article/10.3390/nu13061821/s1, Table S1: The association of risk of malnutrition with low tongue pressure (≤48 kPa).
