# Supplementary material for: Suboptimal Tongue Pressure Is Associated with Risk of Malnutrition in Community-Dwelling Older Individuals
Source: Nutrients. 2021 May 27;13(6):1821. doi: 10.3390/nu13061821 (PMC8229542; doi:10.3390/nu13061821)
Supplement: Supplementary file 1 [file nutrients-13-01821-s001.zip › nutrients-1232943-supplementary.pdf]

**Supplemental Table S1.** The association of risk of malnutrition with low tongue pressure (≤48 kPa).

| **Risk of Malnutrition** | **Maximum Tongue Pressure Separated by the Upper Quartile** | | ***p* Value** |
|---|---|---|---|
| | **Q1-Q3 (*n* = 274) ≤48 kPa** | **Q4 (*n* = 88) (>48 kPa)** | |
| Number (percentage) | 24 (8.75%) | 2 (2.27%) | 0.04 * |
| Model 1 OR (95% CI) | 4.17 (0.96 to 18.04) | 1.0 | 0.05 |
| Model 2 OR (95% CI) | 3.83 (0.88 to 16.65) | 1.0 | 0.07 |
| Model 3 OR (95% CI) | 2.64 (0.58 to 11.82) | 1.0 | 0.20 |

OR, odds ratio. Model 1: no adjustment. Model 2: adjusted for age and sex. Model 3: adjusted for age, sex, grip strength, skeletal muscle index and gait speed. * $p < 0.05$.
